# Hydrophilic Particles Exit While Hydrophobic Particles Persist Following In Vivo Biodegradation of Nanoparticle-Laden Polymeric Devices

**DOI:** 10.1002/anbr.202500005

**Published:** 2025-03-19

**Authors:** Kendell M. Pawelec, Jeremy M. L. Hix, Matti Kiupel, Peter J. Bonitatibus, Erik M. Shapiro

**Affiliations:** Department of Radiology, Michigan State University, East Lansing, MI 48824, USA; Institute for Quantitative Health Sciences and Engineering, Michigan State University, East Lansing, MI 48824, USA; Department of Radiology, Michigan State University, East Lansing, MI 48824, USA; Institute for Quantitative Health Sciences and Engineering, Michigan State University, East Lansing, MI 48824, USA; Department of Pathobiology and Diagnostic Investigation, Michigan State University, East Lansing, MI 48824, USA; Department of Chemistry and Chemical Biology, Rensselaer Polytechnic Institute, Troy, NY 12180, USA; Department of Radiology, Michigan State University, East Lansing, MI 48824, USA; Institute for Quantitative Health Sciences and Engineering, Michigan State University, East Lansing, MI 48824, USA; Department of Biomedical Engineering, Michigan State University, East Lansing, MI 48824, USA; Department of Chemical Engineering and Materials Science, Michigan State University, East Lansing, MI 48824, USA; Department of Physiology, Michigan State University, East Lansing, MI 48824, USA

**Keywords:** clearances, computed tomographies, contrast agents, hydrophobicites, nanoparticles

## Abstract

Longitudinally monitoring biomedical devices postimplantation can improve patient outcomes by allowing targeted intervention during healing. Most polymeric devices are not visible via biomedical imaging technologies. Incorporation of nanoparticle contrast agents into polymer matrices creates imageable devices, but understanding and controlling nanoparticle clearance from the implant site after polymer degradation is needed for clinical translation. To achieve homogeneous distribution throughout biomedical devices, nanoparticle surface chemistry, particularly hydrophobicity, is often manipulated to generate stable suspensions during manufacture. As nanoparticle surface chemistry is a key parameter determining blood circulation, the effects of nanoparticle hydrophilicity on tissue clearance of nanoparticles from implant sites following polymeric device degradation are investigated. Hydrophilic and hydrophobic radiopaque tantalum oxide (TaO_x_) nanoparticles are incorporated at 10 wt% tantalum into gelatin phantoms. In vitro, the diffusion coefficient of released hydrophilic nanoparticles after phantom degradation is significantly greater than hydrophobic nanoparticles, 1.29 ± 0.26 × 10^–5^ and 0.40 ± 0.16 × 10^–5^cm^2^s^–1^, respectively. After subcutaneous implantation in mouse and subsequent phantom degradation, hydrophilic nanoparticles clear skin and muscle tissue within 24 h, whereas hydrophobic nanoparticles remained at the implant site >14 days without change in radiopacity. This clearly demonstrates that nanoparticle surface chemistry must be balanced for initial device manufacturing and final excretion.

## 1. Introduction

Nanoparticle contrast agents are gaining traction in the clinic for their unique properties, such as improved imaging sensitivity and high payloads.^[1]^ While these contrast agents are capable of making anatomical features conspicuous, nanoparticles are also finding use as a way to functionalize medical devices for longitudinal monitoring in a variety of biomedical imaging modalities. The ability to serially track devices postimplantation can enable more timely interventions in patient treatment. It could allow clinicians to diagnose and respond to implant movement, fracture, or premature degradation before patients are adversely affected.

Contrast agents proposed for longitudinal monitoring encompass a wide variety of imaging techniques that are optimized for preclinical to clinical use.^[1]^ Of the available imaging modalities, computed tomography (CT) has a unique advantage in that it is easily transitioned from the lab to the clinic without reoptimizing imaging parameters at each step. In addition, CT is already integrated into clinical workflows and one that is quickly able to generate 3D datasets with high resolution at a relatively low cost.^[2]^ When devices incorporating radiopaque nanoparticles are imaged via CT, a variety of features such as dimensions or pore size can be quantified.^[3]^ Further these features can be tracked noninvasively over time both in vitro and in vivo.^[4–6]^

In creating medical devices that incorporate nanoparticles, it is important to consider how the addition of contrast agent affects the device properties, such as mechanics, as these can be significantly altered.^[4]^ A further complication for biodegradable devices is the necessity to ensure biocompatibility not only of the composite device, but of all breakdown products generated in the body as well. In the case of nanoparticles, after release from a device matrix, they have their own distinct biological response that is a function of their unique properties and the protein corona that forms around them.^[7]^ While studies have been done to understand how nanoparticle size and shape affect the final fate of nanoparticles during excretion, less is known about how nanoparticles clear from tissues other than blood. This has become a barrier to translating radiopaque degradable devices.

An added complication to the incorporation of nanoparticle contrast agents into devices is the requirement for a homogeneous dispersion of the contrast throughout the device.^[3]^ Achieving homogeneity in a composite consisting of a polymer matrix and nanoparticles is heavily influenced by the surface properties of the nanoparticles. In particular, the hydrophobicity of the nanoparticle surface may drive agglomeration or sedimentation of the contrast agent if it is not compatible with the matrix. However, surface properties that are ideal for manufacturing may not encourage efficient clearance within a physiological environment. Indeed, hydrophobicity has been proposed as a major determinant of nanoparticle diffusion through extracellular matrix (ECM).^[8]^ Here, we demonstrate the significant effect of hydrophobicity on the clearance of radiopaque nanoparticles after the degradation of a hydrogel phantom, both in vitro and in vivo. Only hydrophilic nanoparticles cleared tissue effectively, highlighting the necessity to consider and balance nanoparticle excretion when designing novel devices functionalized for biomedical imaging.

## 2. Results and Discussion

An important question on the use of nanoparticles within biomedical devices is the final clearance of these particles from the body and the timescale in which this occurs. Nanoparticle properties, such as size, shape, and hydrophobicity, are known to play important roles in biological excretion.^[7]^ However, the majority of work has been done in systems where nanoparticles or nanomedicines are introduced directly to a circulating a blood pool. When incorporating nanoparticles into biomedical devices, the path of excretion is far more complicated due to interactions with multiple cell types and the dense ECMs of surrounding tissues. Thus, for biomedical devices to incorporate imaging contrast agents, nanoparticle clearance needs to be better characterized in a wider variety of tissues.

### 2.1. Radiopaque Phantoms

Many emerging biomedical devices are based on biodegradable polymers, such as poly(lactide co-glycolide) (PLGA) or collagen.^[9,10]^ When considering the use of biodegradable polymers, the degradation of the device occurs via chain scission of the polymer and diffusion of monomers from the site. In many cases, when nanoparticles are incorporated into devices, it is assumed that the clearance of the nanoparticles and the polymer occur simultaneously during degradation. With nanomedicines, this can be challenging to track, but nanoparticles with inherent biomedical imaging contrast can be monitored over time to measure clearance. In this case, CT-visible tantalum oxide (TaO_*χ*_) nanoparticles were utilized as the imaging agent. They are biocompatible, are relatively low cost compared to noble metals, and have improved imaging enhancement of vasculature over iodinated contrast agents that are constrained for patients with renal issues.^[11,12]^

For in vitro and in vivo testing, the starting amount of nanoparticle contrast agent was kept consistent by embedding the nanoparticles into a gelatin matrix to form a radiopaque phantom. This approach also ensured that nanoparticles were introduced into the animal model within a highly reproducible starting volume. Gelatin was chosen for the matrix for its biocompatibility. It was left uncrosslinked to allow fast degradation under in vivo conditions, focusing on nanoparticle diffusion without the complication of polymer scission kinetics to release nanoparticles into the tissue. The increase in radiopacity of the gelatin phantoms with the incorporation of TaO_*χ*_ nanoparticles was a facile check of nanoparticle loading (Figure 1a).

Given the requirement that contrast agents be incorporated homogeneously throughout a medical device for imaging, the hydrophobicity of the nanoparticles is likely to play a major role in manufacture. Therefore, three types of gelatin phantoms were produced incorporating: 10 wt% hydrophilic nanoparticles, 10 wt% hydrophobic nanoparticles, and a control (0wt% nanoparticles). Both types of particles were based on a TaO_*χ*_ core, with the hydrophobicity determined by the surface chemistry of the particle coating. The hydrophilic particle had a surface coating of a zwitterionic siloxane polymer^[13]^ and was developed as a blood contrast agent. The coating of the hydrophobic nanoparticle was an aliphatic organosilane designed to disperse homogeneously in hydrophobic polymer solutions.^[4]^ Despite the difference in surface coating, all other particle features were kept similar, including the percentage of tantalum (40–52 wt%) and the particle size (4–10 nm). The effect of hydrophobicity on the homogeneity of X-ray attenuation was observed in the gelatin phantoms; hydrophilic nanoparticles imparted a homogeneous X-ray attenuation throughout the phantom, compared to the hydrophobic nanoparticle phantom that showed distinct aggregation of the nanoparticles throughout. Given the propensity for the hydrophobic particles to crash out of solution, the final X-ray attenuation of the devices with hydrophobic nanoparticles was slightly less than in hydrophilic phantoms. The true range of incorporation, after quantification via thermogravimetric analysis (TGA), was found to be between 9.15 and 10.22 wt% tantalum.

X-ray attenuation of nanoparticles demonstrates a linear relationship based on the amount of radiopaque element present.^[14]^ A radiopacity curve for tantalum was constructed in saline, allowing the conversion from X-ray attenuation to the molar concentration of tantalum. It was found that every mM of tantalum increases the X-ray intensity by 5.52 hounsfield units (HU) (28.8 HU per mg Ta mL^–1^), in good agreement with other studies.^[12,14]^ For most in vivo applications, particularly as a blood contrast agent, nanoparticle concentration would be below 200 mM (36 mgTa mL^–1^), often limited by the viscosity of contrast agents during administration.^[13]^ Implanted devices are not necessarily limited in tantalum dose during implantation, and the gelatin phantoms in the current study had an initial tantalum concentration of ≈600 mM (100 mgTa mL^–1^). As Figure 1d shows, the linear range of X-ray attenuation for tantalum nanoparticles extends at least up to 1000 mM (180 mgTa mL^–1^), which provides leeway for tracking nanoparticle concentrations should agglomeration occur at a tissue site over time.

### 2.2. Nanoparticle Diffusion Postdegradation

To validate the rapid degradation of the gelatin phantoms, and the assumption that nanoparticle diffusion after implant degradation would be affected by the particle hydrophobicity, an in vitro study was first performed. Phantoms were placed in saline at 37 °C, and the degradation and subsequent diffusion of nanoparticles were monitored over 3 h using micro-CT (μCT) (Figure 2a,b). As expected for uncrosslinked gelatin, the phantom matrix quickly degraded,^[15]^ forming a concentrated pool of nanoparticles in the bottom of the sample tube.

Utilizing the standard curve for tantalum concentration and radiopacity, the change in X-ray attenuation in the saline buffer was extrapolated to a concentration of nanoparticles over 3 h. After degradation of the phantom, hydrophilic nanoparticles diffused into the saline, leading to an increase in radiopacity of the solution over time, Figure 2c. This corresponded to a decrease in the concentration of TaO_*χ*_ nanoparticles in the concentrated pool as they diffused away. Hydrophobic nanoparticles settled to the bottom of the tube, creating a sharper peak of X-ray intensity (Figure 2d). This confirmed the assumption that the hydrophobicity played a strong role in determining nanoparticle distribution in the aqueous solution under static conditions.

As a measure of the movement of the nanoparticles, diffusion coefficients were determined after 30 min of static diffusion. The measured coefficients were significantly different, 1.287 ± 0.26 × 10^–5^ and 0.400 ± 0.16 × 10^–5^ cm^2^ s^–1^ for hydrophilic and hydrophobic nanoparticles, respectively. This is a higher measured diffusion coefficient than expected for nanoparticles within tissue. Unlike an in vitro saline solution, the in vivo tissue environment is extremely complex. Tissues are composed of a dense network of ECM proteins that can limit the penetration of nanoparticles.^[8]^ Diffusion coefficients through a Matrigel mimic of ECM have been recorded in the order of 10^–8^ cm^2^ s^–1^.^[8]^ The unique composition of ECM in different tissues also has a measurable effect on the diffusion rate of nanoparticles, highlighting the diversity of matrix proteins present in each tissue.^[16]^

### 2.3. Longitudinal Monitoring of Clearance In Vivo

The emerging use of nanoparticles to functionalize biomedical devices and the clear effect of ECM on nanoparticle diffusion necessitated an in vivo study of TaO_*χ*_ nanoparticle clearance. While the majority of nanoparticle contrast agents are designed for use in circulation, clearance of nanoparticles from tissues is far more complicated. Tissues in vivo incorporate interstitial fluid flows, as well as the blood flow through vessels and capillaries. The cellular component of tissues can also complicate nanoparticle clearance as cell surface receptors or tissue resident immune cells interact with nanoparticles.^[17]^ Finally, even after nanoparticles clear from the target tissue, they still have to undergo excretion from the body.

To understand how hydrophobicity impacted clearance in tissue, fast-degrading gelatin phantoms were implanted subcutaneously into mice and monitored longitudinally via μCT over 14 days (Figure 3a). Differences in nanoparticle diffusion through the tissues were almost immediately apparent. Within 30 min, after the degradation of the gelatin phantoms, the hydrophilic nanoparticles spread throughout the muscle adjacent to the implantation site, corresponding to a significant increase in volume compared to the original phantom. By 24 h postimplantation, hydrophilic nanoparticle concentration was below the limit of detection for segmentation from native tissue. In contrast, a portion of the hydrophobic nanoparticles remained close to the implantation site throughout the 14 days, as seen in 2D sections and 3D reconstructions (Figure 3b,c).

In initial time points, the hydrophobic nanoparticles spread into tissue, noted as a slight increase in volume, and then appeared to condense at later timepoints (Figure 3d,e).The movement of nanoparticles through tissue was also recorded by the changes in X-ray attenuation at the implant site. Attenuation dropped significantly upon implantation, and after 24 h, the recorded radiopacity of tissue exposed to hydrophilic nanoparticles was significantly lower than hydrophobic implants. With the large effect size observed at time points after 24 h, a sample size of four had 90% power for discriminating the groups. However, nanoparticle behavior in vivo is influenced by the local biological milieu, with the potential to elicit subtle changes in nanoparticle efficacy and clearance.^[18]^ Thus, to examine clearance from tissues due to effects such as animal age and sex, the sample size will need to increase.

The trends in nanoparticle clearance are consistent with those reported in the literature. When used as a blood contrast agent, hydrophilic nanoparticles have rapid clearance and excretion, with >98% of tantalum cleared within 6 h.^[19]^ The collapse and condensation of incorporated hydrophobic nanoparticles after the degradation of biomedical devices have also been shown previously in other tissues, including in muscle and after implantation in the intraperitoneal space.^[6,20,21]^ When nanoparticles persist within the tissue, they have generally been associated with regions containing a high number of inflammatory cells.^[6]^

A caveat of the use of CT to track nanoparticles is that a minimum concentration of the contrast agent is required to segregate nanoparticles from surrounding tissues. When the concentration falls below this limit, nanoparticles may still be present at the site, suggesting that long-term clearance studies should incorporate additional quantitative measures of nanoparticle concentration, such as inductively coupled plasma mass spectroscopy of surrounding tissues. Contrast agents can be multiplexed to work with multiple imaging modalities, including those that require lower concentrations of contrast agent than CT, like magnetic resonance imaging (MRI).^[22,23]^ However, CT is a low entropy imaging method, producing more predictable information with less complexity, such that signals related to the device are easily distinguished. MRI, on the other hand, has signal intensity changes that are extremely sensitive to tissue physiology, making the discrimination of scaffolds and contrast in situ more complicated, as we have shown previously.^[24]^

Surface properties of nanoparticles apart from hydrophobicity also influence in vivo interaction and diffusion of nanoparticles into tissues. For example, a positively charged coating around nanoparticles facilitated diffusion into negatively charged cartilage lesions.^[25]^ The biological effects due to surface properties are often mediated by the protein corona that develops around nanoparticles once placed into biological fluids. Both tissue clearance and interactions with the immune system are affected by the protein corona. It has been found that the more hydrophobic a nanoparticle coating, the larger the protein corona that attaches and the less protein exchange that occurs within the environment.^[26,27]^ In contrast, a zwitterionic coating, such as those on the surface of the hydrophilic TaO_*χ*_ nanoparticles, shows less protein adsorption due to a creation of a hydration layer that limits protein interactions.^[26]^ In addition, zwitterionic ligands or poly(ethylene glycol) at the surface also decreases cellular uptake by macrophages, thus promoting tissue clearance by hydrophilic particles in this study.^[26]^

The ultimate goal when utilizing tissue engineered biomedical devices is the restoration of tissue function to a healthy state. However, tissue healing is a complex, multistep process. When introducing a foreign material into the body, this process begins with an acute inflammatory phase that generally subsides within a week into a more sustained local foreign body response.^[28]^ The particular action of immune cells and fibroblasts depends on environmental cues and interactions with the implanted material. For example, different components of the immune system upregulate different molecular mechanisms in response to nanoparticle protein coronas.^[29]^ Beyond local reactions to nanoparticles, there is evidence that once nanoparticles reach the circulatory system, they can travel to other organs and trigger low levels of innate immune responses.^[30]^ Thus, when incorporating nanomaterials into devices, it is generally accepted that they should be designed to have low persistence once released from the matrix, to limit the potential cytotoxic effects that can occur over time.

During the study, no adverse effects in the animals were recorded due to the nanoparticles or imaging. The maximal tantalum dosing was 145 mg kg^–1^ when two phantoms with 10 wt% tantalum were implanted on opposite sides of a mouse, which is well within the range of tantalum dosing shown to be toxic to excretory organs.^[12,13]^ It was found that the different types of particles elicited distinct histological patterns during healing, that were again distinct from control implants (Figure 4a–d). Hydrophilic nanoparticles, the majority of which had left the implant site by 24 h, showed a very mild inflammation response at 14 days. In contrast, hydrophobic particles had a prolonged immune response, observed as more severe fibroplasia, granulomatous cellulitis, and ulcerative dermatitis. In all cases, it was difficult to separate the effects of damage from the implantation process and the nanoparticles themselves. The overall immune response was not severe enough to hinder healing.

Given the large role that surface properties play in shaping the protein corona around nanoparticles, it is not surprising that surface properties have been associated to inflammation and toxicity.^[26]^ Nanoparticles with cationic surfaces are generally more cytotoxic than negative or neutral coatings.^[31]^ Also, a hydrophobic surface tends to increase pro-inflammatory marker expression in mouse splenocytes, and even nanoparticle shape can modulate the immune response to nanoparticles.^[32,33]^

In general, the cytotoxicity of nanoparticles in vivo is closely linked with the ability of nanoparticles to be excreted efficiently. There are two main routes for excretion: a hepatic route or the much faster renal route.^[7]^ The pathway nanoparticles take often depends on the size of the nanoparticles, as renal clearance is generally only viable with nanoparticles below 10 nm.^[34]^ However, it is difficult to predict the size of nanoparticles once in the body, as their apparent size changes with the development of their protein corona.^[7]^ In contrast, the hepatic route is mediated both by particle size and by the mononuclear phagocyte system or tissue resident phagocytes. When nanoparticles are of a small size, but elicit a large immune response, the hepatic route can still play a major role in excretion.^[7]^ Through the application of zwitterionic coatings, the immune response can be bypassed in favor of a renal route, if the particles are small enough.^[7,26]^

Importantly, even nanoparticle contrast agents that show effective blood clearance on the order of hours can spend far longer in excretory organs, even out to several months.^[19]^ Questions around nanoparticle behavior and stability after implantation is an active area of research. There is no evidence at this time that the coating of the TaO_*χ*_ nanoparticles will not remain stable, with the zwitterionic coating having been shown to survive autoclaving conditions intact.^[35]^ Regardless of the coating, it is likely that the composition of the protein corona surrounding the nanoparticles will evolve over time.^[18]^ One drawback to metal oxide nanoparticles is their potential to engage in redox reactions, allowing them to act as catalyzing agents in the body. This is particularly true if they are internalized by cells, as large pH ranges exist between cellular compartments.^[18]^ Importantly, tantalum oxide is highly stable, with a dissolution rate of <3% tantalum over 4 weeks at 37 °C, in the pH range of 7–5.5 that covers biological variation from interstitial fluid to the lysosome.^[12]^

The current study highlights significant differences in nanoparticle clearance in a murine model. However, the human biological environment is very distinct from mouse, and the binding of plasma components making the nanoparticle corona is species specific.^[36]^ When comparing the biological response of nanomedicines in tissue, it has been found that clearance is comparable across species, but that the volumes of distribution are significantly higher in humans than other animal models.^[37]^ This effect may be due to the properties of the human protein corona on tissue penetration, higher vascular permeability, greater inflammation, or obesity.^[37]^ In short, the larger distribution volumes make it even more imperative to have effective clearance for nanoparticles to reduce any secondary unwanted biological effects in the clinic. For contrast agents used to functionalize biomedical devices, whatever strategy is utilized to ensure efficient clearance, it is important to consider the effect on imaging contrast and to balance device properties and manufacture with the biocompatibility of degradation products.

## 3. Conclusions

In a growing number of applications, the ability to track implantable polymeric biomedical devices using clinical imaging techniques is proving advantageous for monitoring patient healing. By incorporating nanoparticle contrast agents, polymeric devices can be imaged via a variety of biomedical techniques, which would not be possible otherwise. However, the nanoparticle properties that are optimized for manufacturing devices with homogeneous contrast may not be appropriate for excretion from biological tissue postimplantation. This study found that nanoparticle hydrophobicity has a profound impact on the clearance of nanoparticles. Utilizing μCT, the movement of radiopaque tantalum oxide nanoparticles was tracked after degradation of a gelatin phantom. In vitro, it was confirmed that hydrophilic nanoparticles had greater diffusion coefficients than hydrophobic nanoparticles. Consistent with their use as blood circulating agents, hydrophilic nanoparticles had also cleared muscle and skin 24 h after subcutaneous implantation in a mouse. By comparison, hydrophobic nanoparticles remained at the implant site over 14 days, stimulating a prolonged immune response. This highlights that nanoparticle properties chosen for compatibility with device manufacture must also allow efficient clearance from tissues after implantation. With precision design of nanoparticle coatings and the expansion of manufacturing methods to work with composite materials, there is a bright future for the longitudinal monitoring of biomedical devices.

## 4. Experimental Section

Unless noted, all materials were purchased from Fisher Scientific. For the purpose of this study, nanoparticle hydrophobicity was defined as the ability to be dispersed homogeneously in an organic solvent, while hydrophilicity was the ability to be homogeneously dispersed in an aqueous solution.

### TaO_χ_ Nanoparticles:

Hydrophobic TaO_*χ*_ nanoparticles were coated with an aliphatic organosilane, based on our previous procedure.^[12]^ To ensure dispersion in organic solvents, the organosilane used was (3-aminopropyl) trimethoxy silane (Sigma). The TaO_*χ*_ nanoparticles were spherical, with a diameter range of 3–9 nm.

Hydrophilic nanoparticles were formulated as previously described (240 mg of tantalum mL^–1^).^[35]^ This concentrated aqueous nanoparticle solution was desiccated at 70 °C and then ground to a fine powder of particles by mortar and pestle for incorporation into the phantoms.

### Phantom Manufacture:

Phantoms were constructed from gelatin (Type A from porcine skin, Sigma G2625), to contain 10 wt% gelatin and 10 wt% tantalum. Solutions of 10 wt% gelatin were first heated at 70 °C until the gelatin was completely dissolved and clear. The solution was then filtered through a 0.2 μm filter to ensure sterility. Preweighed tantalum nanoparticles were exposed to UV light for 30 min in a biological fume hood and then added to the gelatin solution and vortexed. All solutions were left at 70 °C for an additional 15 min until casting. Just before casting in silicon molds, solutions were vortexed again briefly. Each mold was filled with 40 μL of gelatin solution and allowed to gel at room temperature in a sterile environment before removal from the mold. Samples were stored at room temperature until final use.

### TGA:

To quantify the weight percent of TaO_*χ*_ in the phantoms, TGA was utilized. The time from manufacture to testing was minimized to ensure low loss of water due to gel drying. For each phantom type, 20–25 mg of phantom material was massed in a platinum pan. Heating was performed at a rate of 10 °C min^–1^ to 600 °C under a nitrogen environment using a TA Q500 (TA Instruments). The percentage of nanoparticles was calculated as the difference between the initial and final sample mass. All calculations were normalized by the mass of the control (10 wt% gelatin + 0 wt% nanoparticles) to account for incomplete removal of ash during the measurement.

### Nanoparticle Diffusion:

To observe the diffusion of nanoparticles in an in vitro environment, phantoms containing nanoparticles were serially imaged via μCT over 3 h. To emulate an in vitro environment as closely as possible, degradation was conducted in phosphate saline buffer (PBS) at 37 °C. Prewarmed microcentrifuge tubes containing 0.5 mL PBS were imaged (time = –1 min) prior to starting the experiment. Phantoms were placed in individual tubes and imaged immediately (time = 0 min). After the initial time point, phantoms were then imaged every 5 min, up to 30 min and again at 60, 120, and 180 min. In between imaging, tubes were carefully removed to avoid agitation and kept at 37 °C. The intensity of the PBS was recorded over time, details in Section 4.6. Each diffusion study was conducted with *n* = 3 samples.

In addition to tracking tantalum concentration over time, a diffusion coefficient for each nanoparticle type was calculated from the diffusion study. Using ImageJ, the intensity profile of the sample was plotted as a function of the distance from the bottom of the tube (or source of nanoparticles) at time = 30 min. The resulting profile was smoothed by averaging with 10 points on either side, and fit to the following equation for 1D diffusion of a plane^[38]^ using a nonlinear fit function in GraphPad Prism (v. 10.4.0).


(1) $$C(x,t)=C_{0}\exp\;\left(-\frac{X^{2}}{4Dt}\right)$$

where *t* is time of diffusion (seconds), *x* is the diffusion distance (cm) and *C*_o_ is the initial concentration of nanoparticles at *x* = 0 (in M). The fit was used to find the diffusion coefficient (*D*) in each sample, and an average of three samples is reported for each nanoparticle type.

### μCT:

A Revvity Quantum GX was used for all biomedical imaging. To determine nanoparticle diffusion, phantoms in PBS were imaged at 90 keV, 88 μA, with a 25 mm field of view. This led to a voxel size of 50 μm. A radiopacity curve was constructed to relate measured X-ray attenuation to tantalum concentration. Each concentration of tantalum was made up of hydrophilic nanoparticles suspended in PBS, to directly compare with the buffer used for phantom degradation. Two independent repeats of the curve were run, from 0 to 1 M tantalum, and scanned under the same conditions as phantoms.

In vivo μCT on mice was also performed at 90 keV, 88 μA, as previously reported.^[6]^ Scans of mice prior to implantation served as a baseline for tissue radiopacity. These scans were taken at a 36 mm field of view (4 min scan time) at 72 μm resolution. Postimplantation, scans were taken using a 72 mm field of view (4 min total scan time) at 90 μm resolution. These parameters were chosen, as they allowed visualization of both phantoms in a single scan. Scanning time points postimplantation were at 30 and 180 min on day 0 and then on days 1, 3, 7, and 14. Total cumulative radiation dosage was 3.92 Gy over 2 weeks. Of the total dose, the baseline scan (2.95 Gy) exposed only a local region around the implant site to radiation, while the remaining 0.97 Gy scanned a larger region. This radiation dose is well below the mouse LD 50/30.^[39]^

### μCT Image Analysis:

From the μCT scans of in vitro phantoms and radiopacity curves, X-ray attenuation was quantified using Image J. Each individual measurement of intensity reported for a given time point and location were averaged from at least five slices through the sample image stack to get a representative value. To construct a radiopacity curve, measured values, normalized to the blank buffer, were fitted with a linear regression model. For measuring changes in X-ray attenuation in time lapse images, areas of 1 mm thickness were isolated in the sample media starting from *x* = 0 (the base of the microcentrifuge tube) and then at 4, 6, and 16 mm. Values of intensity were normalized by the attenuation of the buffer alone (from the *t* = –1 min scan). The normalized intensity was then converted to a molar concentration of tantalum based on the radiopacity curve. All data quantified from μCT scans is reported as mean ± standard deviation.

To quantify the radiopacity and volume of phantoms postimplantation, the ITK-SNAP was utilized.^[40]^ The volume of tissue containing nanoparticles was segmented from the background tissue using built in segmentation tools. Regions with nanoparticles were identified by increased X-ray attenuation. The volume of the tissue segmented and its average intensity was calculated for each time point. Hydrophilic nanoparticles could not be accurately segmented after 180 min scans, and thus the reported X-ray attenuation is the value of soft tissue for the entire implant region. To create 3D reconstructions from the in vivo scans, Dragonfly (v.2022.2) built in functionality was utilized. All data quantified from μCT scans was reported as mean ± standard deviation.

### Phantom Implantation:

All procedures were performed in accordance with IACUC approved protocols and Veterinary guidelines at Michigan State University (PROTO202100337). FVB mice (*n* = 6 adult male and female, >9 months old; Charles River Laboratories) were used for this surgical implantation and μCT imaging study. Each mouse was surgically implanted with two gelatin phantoms containing either 0 or 10 wt% TaO_*χ*_ nanoparticles (hydrophilic or hydrophobic) installed on opposite sides of the animal. Sterile phantoms, 4.7 mm diameter and 1.5 mm thick, were prepared on the day of implantation. A sample size of four had 90% power to cover the large effect sizes observed.

The gelatin phantoms were implanted subcutaneously. Each animal was administered analgesia at least 20 min prior to making the initial incision, as detailed previously.^[6]^ Local infiltration of 2% Lidocaine (diluted to 0.5%) was administered subcutaneously (S.C.) along the intended incision site just prior to making the incision (7 mg kg^–1^ max dose; SID). Mice were anesthetized and maintained via inhalant isoflurane during surgery (1–3% isoflurane in 0.8–1 LPM oxygen), with supplemental heat via recirculating warm water blankets. To implant the phantoms, a subcutaneous pocket was created, and a phantom was placed inside before closing the site using Vetbond tissue glue (N-butyl cyanoacrylate). Following animal recovery, meloxicam (5 mg kg^–1^; SID) and Ethiqa XR Buprenorphine Sustained Release (3.25 mg kg^–1^, SID, once) were administered S.C. for 72 h postsurgery. Postoperative clinical observation, body weight assessment, and health score assessments were performed for 7–14 days postoperatively. At termination, animals were sacrificed via CO_2_ overdose asphyxiation.

### Statistics:

Statistics were performed using GraphPad 10.4.0. Where data was fit using linear and nonlinear models, *R* squared values were maintained above >0.95. Groups were compared via analysis of variance, followed by Bonferroni’s multiple comparisons test. In all cases, *α*< 0.05 was considered significant, with a 95% confidence interval.

## Figures and Tables

**Figure 1.**
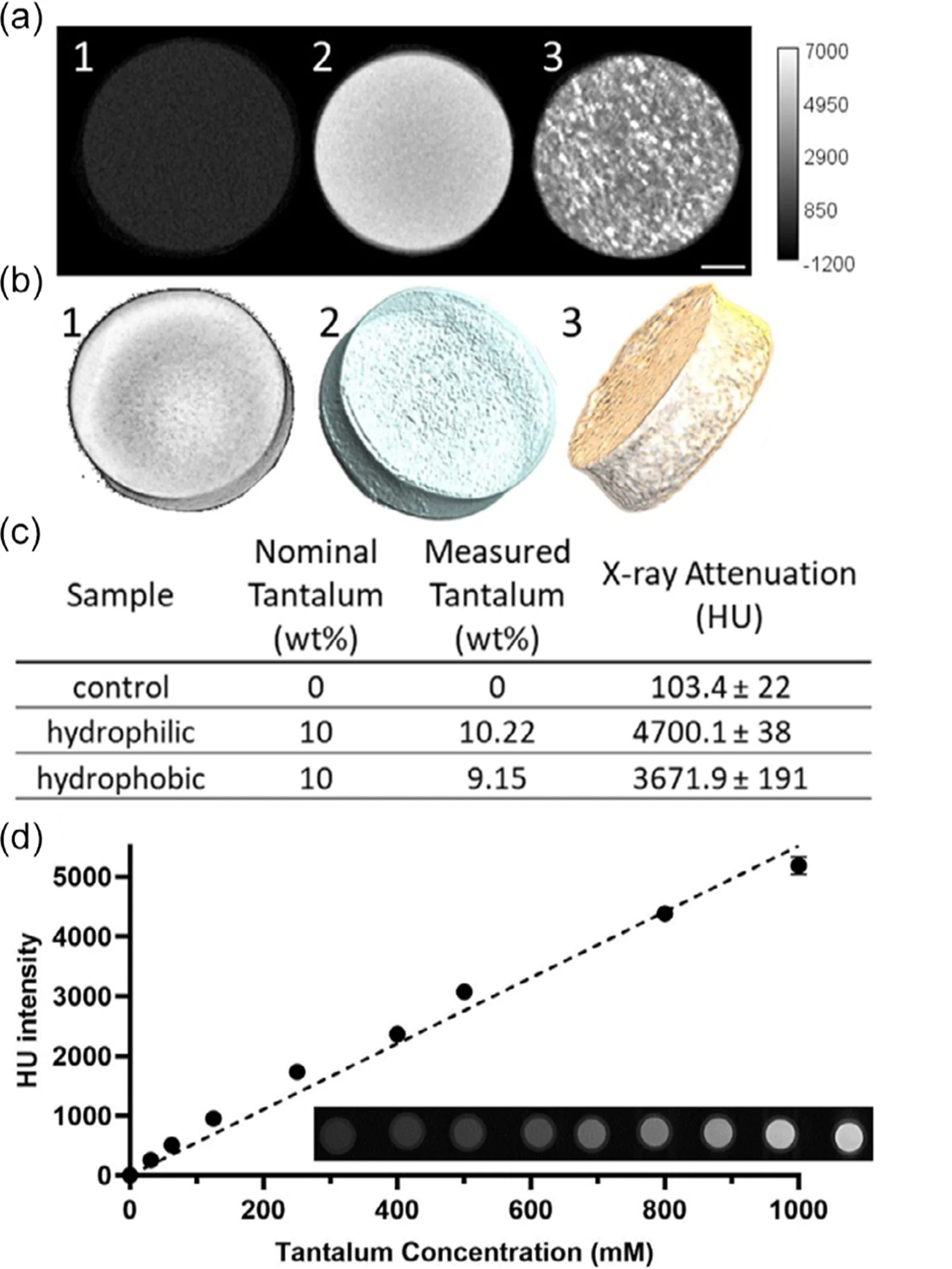
Radiopaque phantoms were produced by embedding radiopaque TaO_*χ*_ nanoparticles in 10 wt% gelatin. The phantoms were visualized by a) μCT with b) 3D rendering demonstrating similarity of phantom size and shape of: (1) 0 wt% Ta, (2) 10 wt% hydrophilic Ta, and (3) 10 wt% hydrophobic Ta. c) Tantalum content was close to nominal values and dictated the X-ray attenuation of the phantoms. d) A linear relationship was observed between tantalum concentration and X-ray attenuation up to 1 M Ta concentration. Scale bar (a) 1 mm.

**Figure 2.**
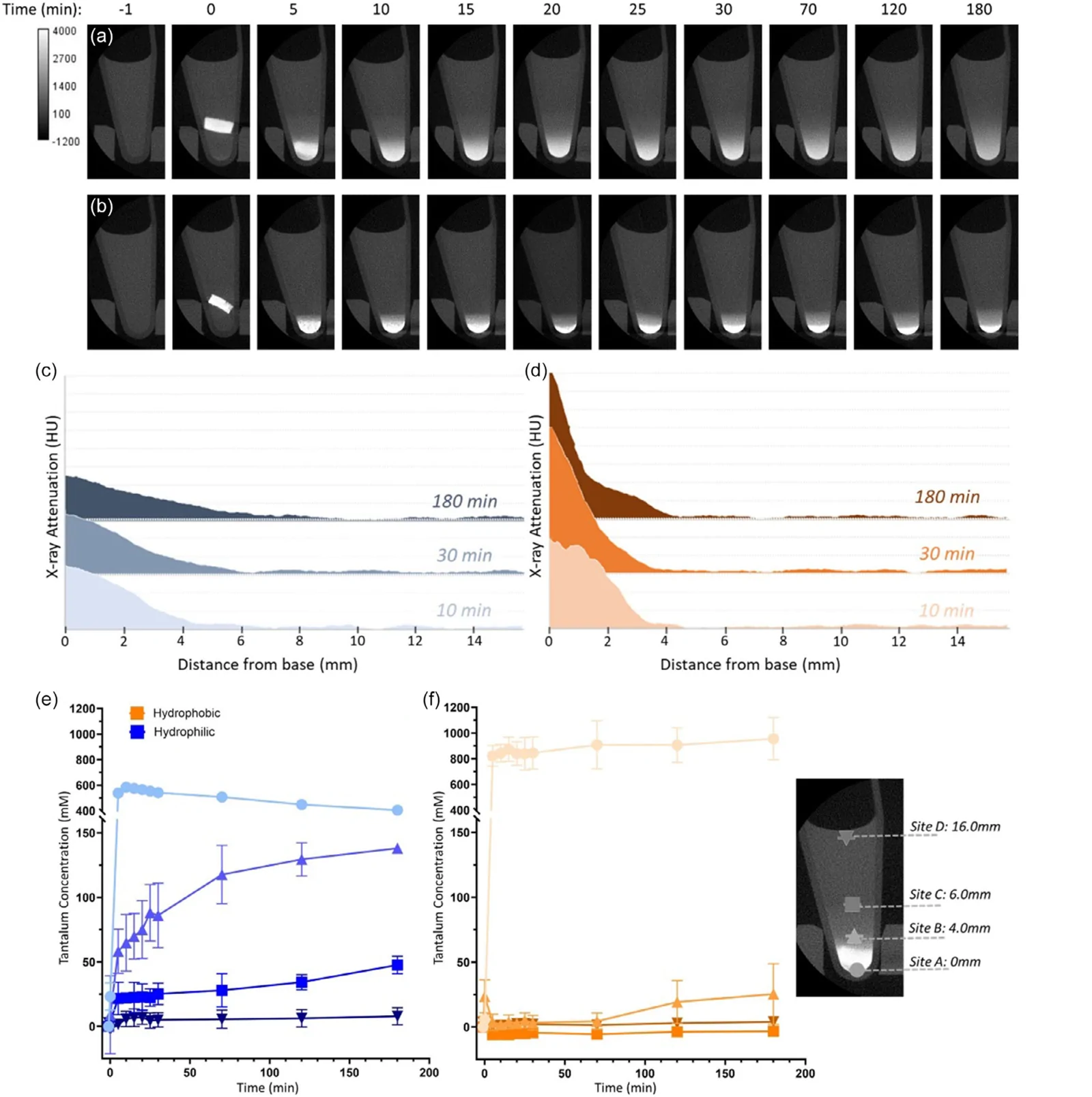
After device degradation, hydrophobicity determined the diffusion of nanoparticles in saline buffer, as seen in serial monitoring of particles via μCT for a) hydrophilic nanoparticles and b) hydrophobic nanoparticles. The X-ray attenuation through the sample volume, and therefore the tantalum concentration, evolved over time for c) hydrophilic nanoparticles and d) hydrophobic nanoparticles. Intensity curves are stacked to show their evolution; each line on the y-axis is separated by 1000 HU. e,f) The tantalum concentration at various points in the volume, as shown in the inset, were quantified over time. (e) After phantoms with hydrophilic nanoparticles degraded, diffusion away from the concentrated source began. (f) In hydrophobic phantoms, particles settled and compacted over time in the solution. Scale bar (a,b): 1 mm; HU window is consistent throughout the figure.

**Figure 3.**
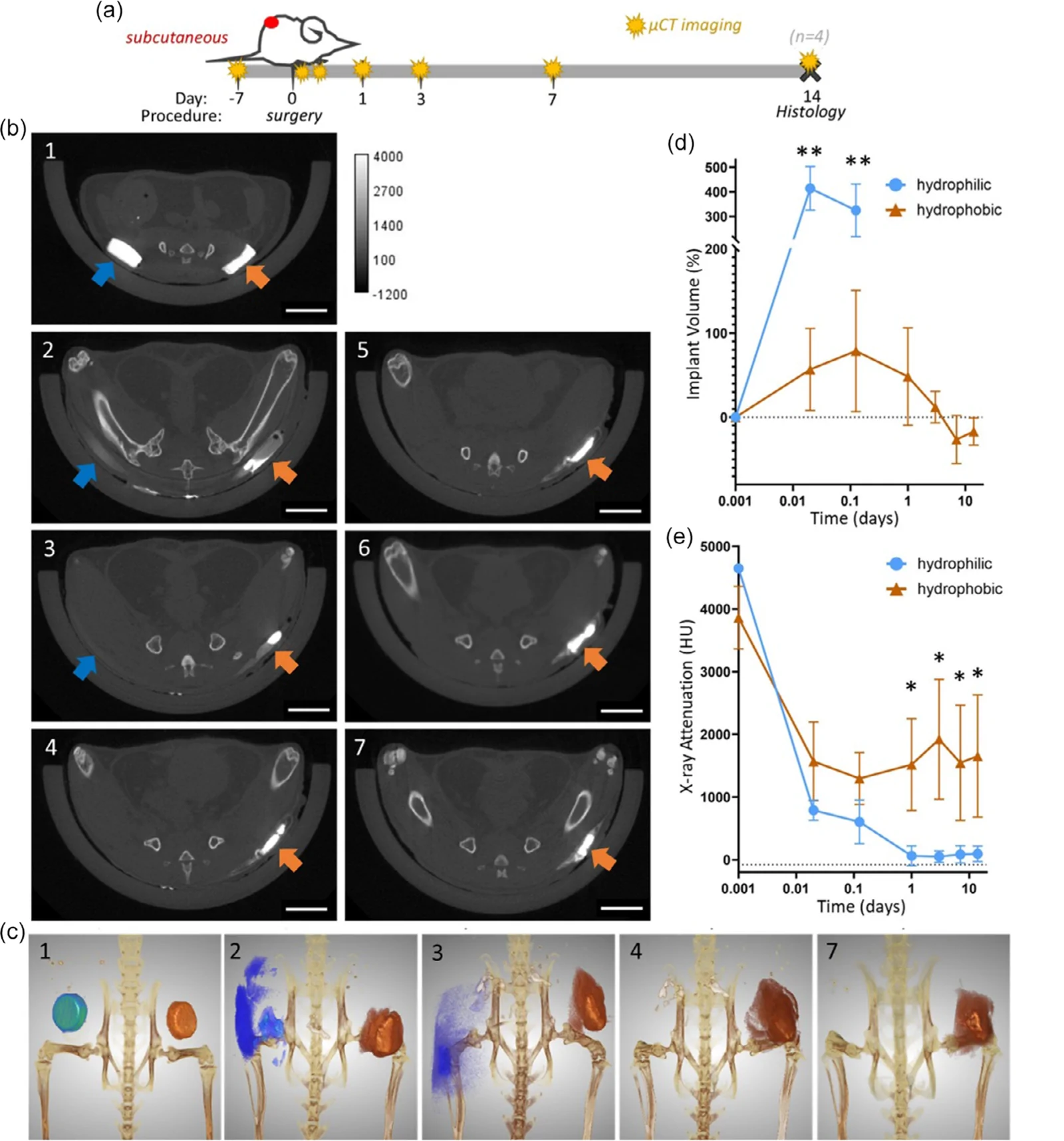
Tissue clearance of nanoparticles in vivo depended strongly on the surface hydrophobicity of the TaO_*x*_ nanoparticles. a) Outline of the animal experiment and imaging time points. b) At each time point, μCT images allowed the tracking of nanoparticles as they diffused after phantom degradation, highlighted by c) 3D renderings of the devices. Where visible, devices with hydrophilic nanoparticles are marked with blue arrows and hydrophobic with orange for (1) day 0, 0 min; (2) day 0, 30 min; (3) day 0, 180 min; (4) day 1, (5) day 3, (6) day 7, and (7) day 14. d) The volume of the tissue with nanoparticles was tracked over time and normalized by pre-implant volume. e) X-ray attenuation of the implant was also quantified where an implant was able to be segmented from background tissue or, alternatively, the X-ray attenuation of the tissue around the implant site was recorded. After 24 h postimplantation, the remaining hydrophilic nanoparticles could not be segmented. Data reported as mean ± standard deviation (*n* = 4). Significantly different from initial values: *p < 0.01, **p < 0.0001. Scale bar (b): 5 mm.

**Figure 4.**
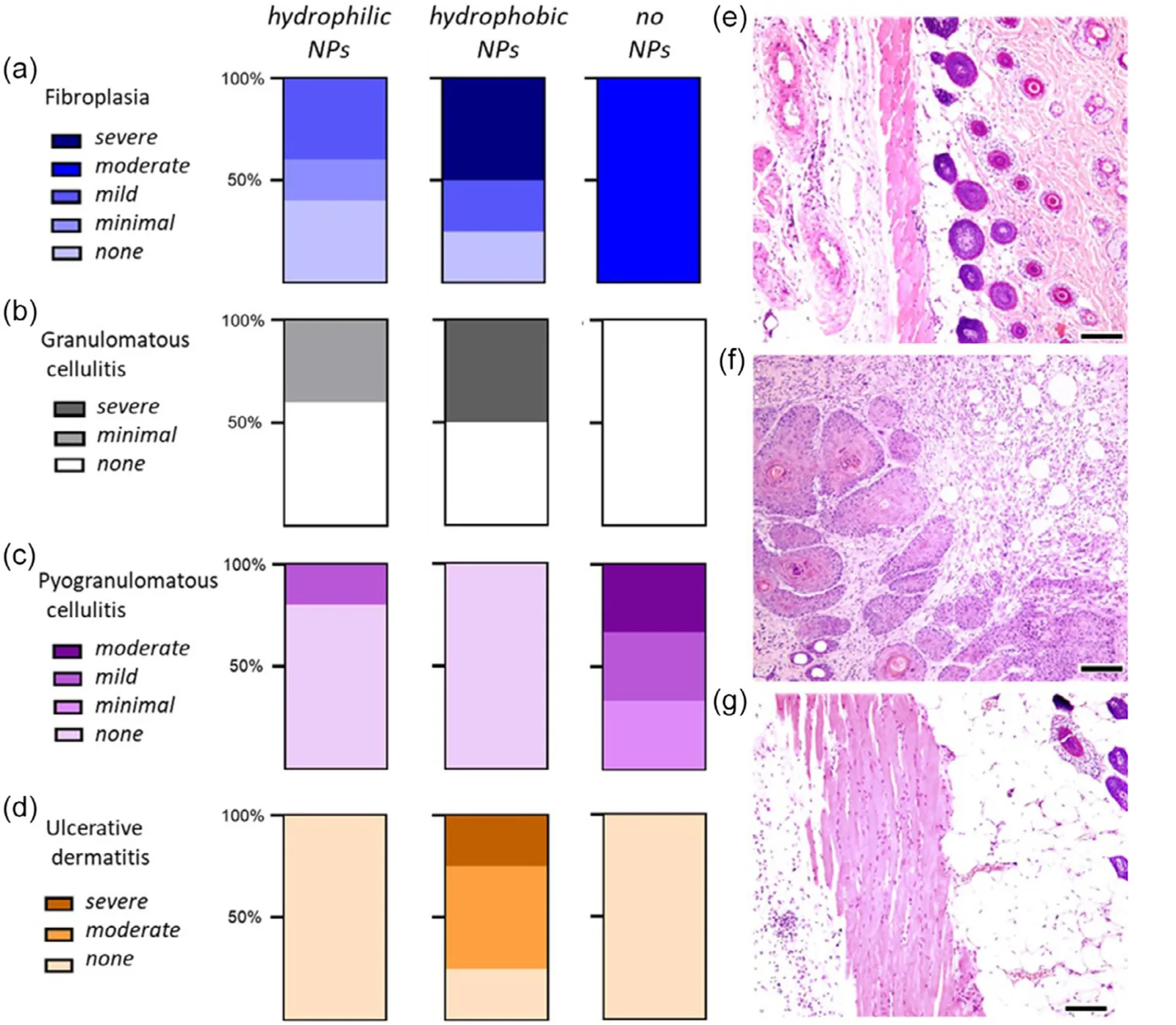
Inflammation at the implant site was influenced by the hydrophobicity of nanoparticles. In all cases, an immune reaction was observed postimplantation of devices consisting of a) fibroplasia, b) granulomatous cellulitis, c) pyogranulomatous cellulitis, and d) ulcerative dermatitis. Representative histology of the skin around the implant site after 14 days is shown for e) hydrophilic nanoparticle devices, f) hydrophobic nanoparticle devices, and g) control devices (0 wt% nanoparticles). Scale bar (e–g): 100 μm.

## Data Availability

The data that support the findings of this study are available from the corresponding author upon reasonable request.
